# Specific tracking of xylan using fluorescent-tagged carbohydrate-binding module 15 as molecular probe

**DOI:** 10.1186/s13068-016-0486-1

**Published:** 2016-03-25

**Authors:** Vinay Khatri, Yannick Hébert-Ouellet, Fatma Meddeb-Mouelhi, Marc Beauregard

**Affiliations:** Centre de recherche sur les matériaux lignocellulosiques, Université du Québec à Trois-Rivières, C.P. 500, Trois-Rivières, QC G9A 5H7 Canada; PROTEO, Université Laval, Quebec, QC G1V 4G2 Canada; Buckman North America, Vaudreuil-Dorion, QC J7V 5V5 Canada

**Keywords:** Carbohydrate-binding module, Fluorescent protein, Kraft pulp, X-ray photoelectron spectroscopy, Xylan, Xylanase

## Abstract

**Background:**

Xylan has been identified as a physical barrier which limits cellulose accessibility by covering the outer surface of fibers and interfibrillar space. Therefore, tracking xylan is a prerequisite for understanding and optimizing lignocellulosic biomass processes.

**Results:**

In this study, we developed a novel xylan tracking approach using a two-domain probe called OC15 which consists of a fusion of *Cellvibrio japonicus* carbohydrate-binding domain 15 with the fluorescent protein mOrange2. The new probe specifically binds to xylan with an affinity similar to that of CBM15. The sensitivity of the OC15-xylan detection approach was compared to that of standard methods such as X-ray photoelectron spectroscopy (XPS) and chemical composition analysis (NREL/TP-510-42618). All three approaches were used to analyze the variations of xylan content of kraft pulp fibers. XPS, which allows for surface analysis of fibers, did not clearly indicate changes in xylan content. Chemical composition analysis responded to the changes in xylan content, but did not give any specific information related to the fibers surface. Interestingly, only the OC15 probe enabled the highly sensitive detection of xylan variations at the surface of kraft pulp fibers. At variance with the other methods, the OC15 probe can be used in a high throughput format.

**Conclusions:**

We developed a rapid and high throughput approach for the detection of changes in xylan exposure at the surface of paper fibers. The introduction of this method into the lignocellulosic biomass-based industries should revolutionize the understanding and optimization of most wood biomass processes.

**Electronic supplementary material:**

The online version of this article (doi:10.1186/s13068-016-0486-1) contains supplementary material, which is available to authorized users.

## Background

Lignocellulosic biomass is a major source of sugars for the production of biofuel [1, 2]; however, its production has always been hindered by several economical and technical obstacles [3, 4]. One of these obstacles is the complex structure of the lignocellulosic substrate. As a consequence, the enzymatic hydrolysis of lignocellulosic components to fermentable sugars is considered as one of the major rate-limiting and costly steps [3, 5–8]. One way to better understand and control hydrolysis of lignocellulosic biomass is to monitor the complex polymers composition of fibers at every stage of processing.

Lignocellulosic biomass is a complex structure consisting of cellulose (β-1,4-linked glucose polymer), hemicellulose (polysaccharide of varying compositions), and lignin [9]. Cellulose is the most abundant polysaccharide in nature and constitutes about 35–50 % of the total lignocellulosic biomass [10]. Hemicelluloses, which represent about 20–30 % of the total biomass, are the second most common polysaccharides [10, 11]. Unlike cellulose, hemicelluloses are heterogeneous polymers of pentoses (xylose, arabinose), hexoses (mannose, glucose, galactose), and/or uronic acids (glucuronic acid, galacturonic acid) [9, 12]. Hemicellulose in softwood (from gymnosperms) contains mostly glucomannans whereas in hardwood (from angiosperms) mostly consists of xylan [13]. The hemicelluloses distribution on the surface of wood fibers/cellulose fibrils is of utmost importance for the complex structure of lignocellulosic biomass, since hemicelluloses have been proposed to act as a physical barrier which increases the stiffness of the cellulose fiber network by coating the rigid cellulose crystallites and forming links between the fibrils [3, 14].

Among hemicelluloses, xylans are the most abundant and complex hemicelluloses comprising a backbone of β-1,4-linked xylopyranosyl residues [10, 11, 13]. Xylan has been shown to limit the accessibility of cellulase enzymes to cellulose [15–20]. As a consequence, biomass bioconversion requires the presence of accessory enzymes such as xylanase, which allows for controlling the significant effect of residual xylan on cellulose accessibility during bioconversion [4]. The cost associated to enzyme utilization is an important aspect of bioenergy production. [15, 21–23]. Enzymes cost can be minimized by tighter control of process parameters (such as dosage and incubation time). To this end, one needs to track lignocellulosic polymers, including xylan, at various stages of processing.

Pulp and paper production is another lignocellulosic biomass-based industry which has to deal with the complexities described above. In addition, this industry faces immense pressure from the society and/or governments to move toward green chemistry. Biocatalysts are recognized as a key element of green chemistry and are being progressively introduced in a number of processes with extremely positive consequences for the environment [24, 25]. An increasing number of enzymatic strategies are used by paper makers, including the application of xylanase enzymes in the pre-bleaching or bio-bleaching of kraft pulp. The presence of xylan, and its redeposition on the surface of cellulose fiber during the kraft pulping of hardwood, inhibits the bleaching process. Xylanase enzymes have been found to be most effective for limiting this problem and are now in use at several mills worldwide for bio-bleaching [13, 15, 24–26]. Further, xylan is also known to contribute to fiber strength and its removal is known to influence pulp fiber properties [15, 27, 28]. Xylan is believed to contribute to physical properties of the paper by enhancing the inter-fiber bonding [27]. Here again, the close monitoring of xylan would help optimize the enzymatic treatment, better control of paper properties, and minimize its cost.

In order to make these lignocellulosic biomass-based processes highly productive and cost-effective while improving quality of end-products, one should correlate the process parameters (such as enzyme loading, temperature, or treatment time) to the substrate availability in a given biomass sample, or to a given percent removal target (*i.e.*, × % decrease in xylan at the surface of cellulose fibers). Unfortunately, current methods for tracking xylan are not compatible with industrial constraints. To date, tools such as X-ray photoelectron spectroscopy (XPS or ESCA) [29, 30], atomic force microscopy (AFM) [31], scanning electron microscopy (SEM) [30], time-of-flight secondary ion mass spectrometry (ToF–SIMS) [30], gas chromatography (GC) [32], Fourier transform infrared spectroscopy (FTIR) [33], and chemical methods [34, 35] have been used to study the surface and bulk chemistry of wood fibers. However, use of these methods for lignocellulosic biomass analysis is laborious, requires specialized equipment, tedious sample preparation, and long analysis time (typically hours for each sample) [36, 37]. As a result, it is highly challenging to tightly modulate the amount of xylanase used for complete or selective xylan removal for process optimization.

Over the past decade, other techniques have been developed for the direct and rapid detection of lignocellulosic biomass polymers. The use of chemical dyes to stain lignocellulosic biopolymers was one of the initial approaches for the detection of cellulose within various materials. Unfortunately, these dyes are rarely specific to cellulose [38]. In recent years, several in situ detection techniques have been developed, not only for cellulose but also for other cell wall components, including hemicellulose and pectic polysaccharides detection [39]. Among these techniques, monoclonal antibodies (mAbs) have been used successfully for developmental studies of vegetal materials. However, antibodies targeting complex polysaccharides, made of crystalline and insoluble structures, are difficult to generate [38, 40]. Like antibodies, carbohydrate-binding modules (CBMs) are highly specific toward their substrate polysaccharides. They have been shown to discriminate crystalline cellulose from amorphous cellulose [38, 40].

CBMs are the non-catalytic polysaccharide-recognizing module of enzymes such as glycoside hydrolases [41–43]. CBMs play a central role in the optimization of the catalytic activity of plant cell wall hydrolases by their specific binding to plant polysaccharides. These CBMs are grouped into 71 different families, based on amino acid sequence homology, in the Carbohydrate Active enZymes (CAZy) database (http://www.cazy.org/) [41]. CBMs are further classified into three types A, B, and C, on the basis of three-dimensional structure and functional similarity. Type A CBMs recognize the surface of crystalline cellulose, type B and type C CBMs are identified as CBMs that recognize internal glycan chain (*endo*-type) and terminal (*exo*-type) glycans, respectively [43, 44]. Among type B CBMs, the family 15 CBM (CBM15) includes the non-catalytic xylan-recognizing module of a xylanase (Xyn10C from *Cellvibrio japonicus)* which has been demonstrated to bind xylan, including substituted xylan and xylooligosaccharides [45]. The high specificity of CBMs toward lignocellulosic polymers makes them more interesting as probes compared to mAbs [38, 40, 41]. CBMs have been used for several applications related to biomedicine, environment, molecular biology, microarrays, paper, textile, food, and biofuel industries [41]. Considering the importance of xylan detection for industrial processing of lignocellulosic biomass, we propose to use nature’s own recognition molecules (CBMs) as the spearhead of an efficient xylan detection method.

Fluorescence is a very sensitive and specific spectroscopy where absorption and emission wavelengths determine what molecules contribute to the detected signals [43, 46]. Further, plate readers allow increasing measurement throughput, a valuable criterion in the development of any novel assay. Hence, detection of CBM probes that would emit fluorescence would be advantageous. Fluorescence detection can be achieved directly or indirectly depending on the methods used [38]. The indirect methods involve the use of a secondary or tertiary reagent such as anti-His-IgG coupled to a fluorophore to detect the His-tag of a CBM, which may also allow amplification of signal intensities. This method provides great flexibility in CBM use but has a potential disadvantage related to multi-step incubations which decrease analysis speed and are less compatible with a high throughput strategy [38]. On the other hand, in direct methods, coupled CBMs would require a straightforward, single-step incubation, affording the possibility of rapid, high throughput protocols. In the first direct method reported, a CBM was chemically coupled with a fluorophore (such as FITC/Alexa Fluor) [38]. Unfortunately these molecules react non-specifically with various moieties at the surface of CBMs, deleteriously impacting specificity, affinity, and detection reproducibility. Another direct detection method uses CBMs as fusion with a fluorescence protein such as the green fluorescent protein (or any of its variants) [38]. This method allows maintenance of the original CBM behavior, avoiding the limitations described for the first direct method discussed. Hence, CBMs coupled with fluorescence protein have been used for mapping the chemistry and structure of various carbohydrate-containing substrates (lignocellulosic biomass) [47, 48]. More recently, two different recombinant fluorescent CBM probes have been used for quantitative study of the change of accessibilities of amorphous cellulose and crystalline cellulose regions during the enzymatic hydrolysis of Avicel [43].

In this study, we demonstrate the potential of a fluorescent-tagged fusion protein mOrange2-CBM15 probe (hereafter named OC15) for monitoring xylan at the surface of paper samples. To evaluate the potential of our novel method, we decided to use two different grades of kraft pulps (unbleached and bleached), and we analyzed xylanase-treated pulp in order to study the sensitivity of the developed method. Our results suggest that such probes can form the basis of a rapid, easy to use, unambiguous and affordable diagnostic approach, helping optimizing treatment strategy, and reducing the cost of processes which rely on controlled xylan removal.

## Results and discussion

### OC15 expression and purification

**Fig. 1:**
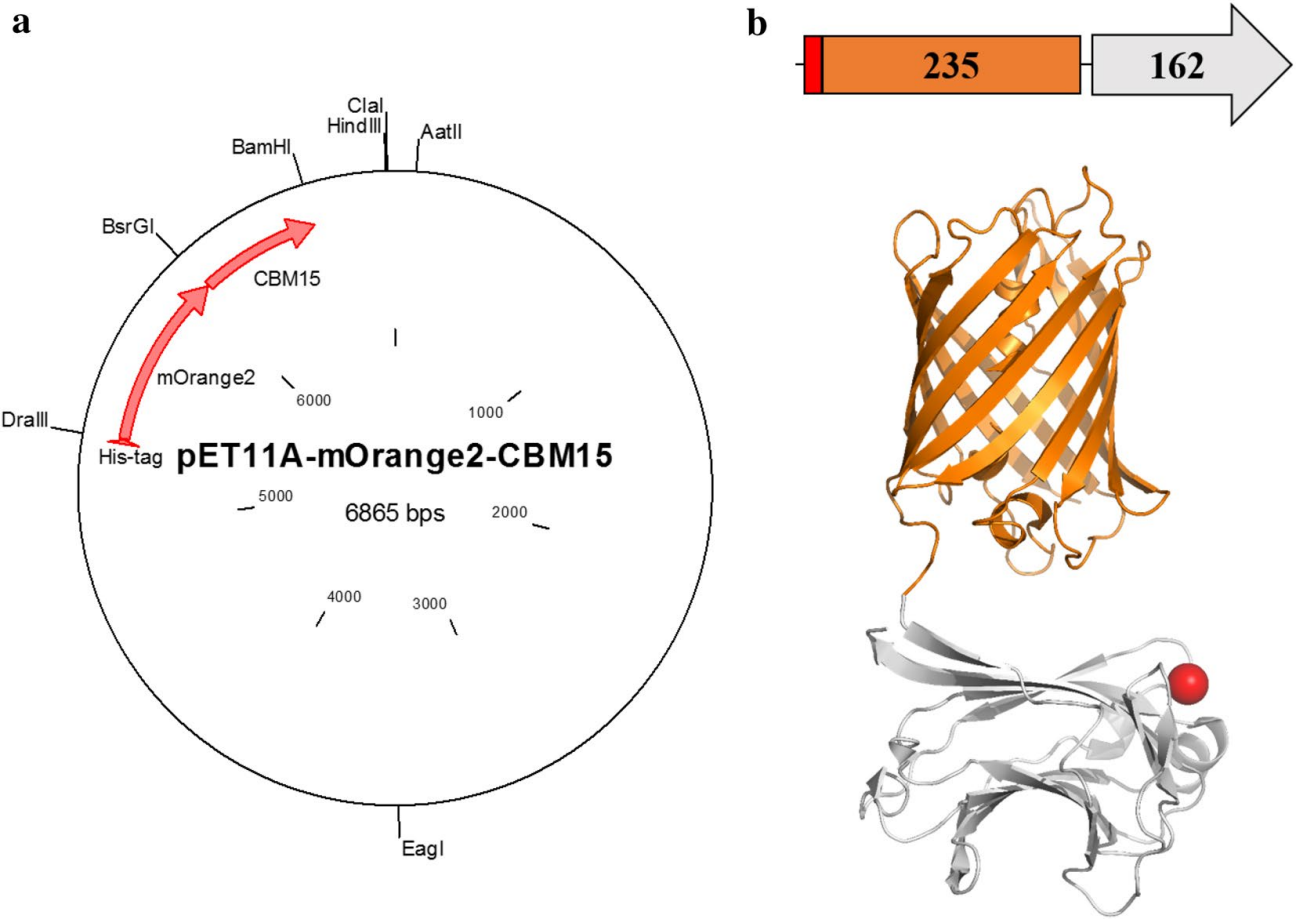
Plasmid map (**a**), construction *scheme* and *pictorial* representation (**b**) of the OC15 probe. mOrange2 C-terminal end is linked to the N-terminal end of CBM15 (*Cellvibrio japonicus*). The *red rectangle* represents N-terminal six histidines tag. The *red sphere* represent the metal ion in CBM15. The *structural model* shown was constructed using PDB files 1GNY and 2H5O (obtained for the closely related mOrange fluorescent protein). The sequence linking the fluorescent protein to the CBM is composed of a glycine residue

A two-domain recombinant probe named OC15 was designed for specific tracking of variations of xylan on the surface of lignocellulosic material (Fig. 1). *Cellvibrio japonicus* CBM15 composed the xylan recognition domain (C-terminal) while monomeric fluorescent protein Orange2 constituted the probe detection domain (N-terminal). OC15 was expressed in *E. coli* BL21-Gold(DE3)pLysS cells which contained the pET11a-mOrange2-CBM15 plasmid (Fig. 1a, b). The expected molecular weight of OC15 is 44.68 kDa. Following affinity and size exclusion chromatography steps, the probe purity was verified using SDS-PAGE (Additional file 1). Interestingly, the gel analysis of OC15 revealed two bands: one intense band, corresponding to OC15 expected size (44.68 kDa), and another, less intense band (less than 1 % on the basis of staining intensity) of a smaller size. A similar result has been observed for the purified mCherry-CBM17 probe designed by Gao et al. [43]. These authors showed that the smaller band was the result of an incomplete denaturation of the probe under standard SDS-PAGE conditions. We investigated this possibility and found that increasing the SDS concentration in the gel, sample, and running buffers decreased the intensity of the smaller band (data not shown). Therefore, we concluded that, like the probes of Gao et al., the OC15 probe is incompletely denatured under standard SDS-PAGE conditions.

### Determination of OC15 ligand specificity using affinity gel electrophoresis (AGE)

**Fig. 2:**
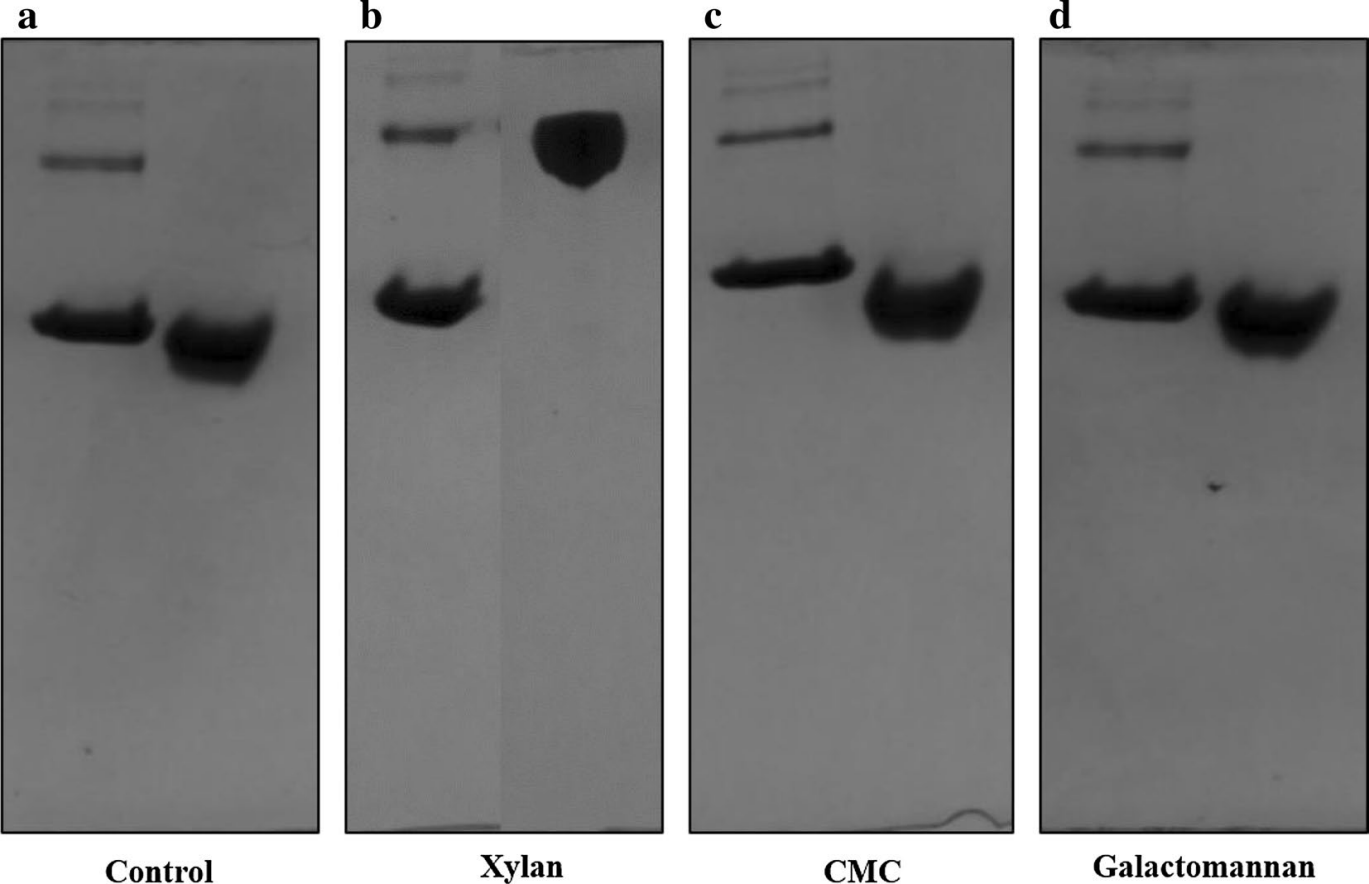
Affinity gel electrophoresis (AGE) of OC15 probe. **a** Control (no polysaccharide), **b** xylan, **c** CMC, **d** galactomannan. In *each* *panel* the first well contained BSA (10 µg) and the second well was loaded with OC15 probe (10 µg). All soluble polysaccharides were used at final concentration of 0.5 % (w/v) and a 12 % polyacrylamide gel was used for affinity analysis

Affinity gel electrophoresis (AGE) was used to qualitatively evaluate the specificity of the OC15 probe toward soluble polysaccharides [49]. Interaction between the studied protein and the gel-embedded polysaccharide is revealed by a reduced mobility compared to the mobility of the protein in absence of saccharide. BSA, which has no affinity toward carbohydrates, was used as negative control [49]. Figure 2 shows that OC15 interacts only with beechwood xylan (Fig. 2b). Similarly to BSA, no binding was detected between OC15 and carboxymethyl cellulose (Fig. 2c) or galactomannan (Fig. 2d). These results confirm that the well-known specific binding to xylan of CBM15 is unaltered by its fusion with mOrange2 in the OC15 probe. However, the affinity of the recognition module of the OC15 must still be determined in order to ascertain its ability to sensitively detect xylan on the surface of paper.

### Determination of OC15 ligand affinity using isothermal titration calorimetry (ITC)

The affinity of OC15 toward hexaoses was also investigated by ITC (Table 1; Additional file 2). Analysis of the binding isotherms showed that the recognition module of the OC15 probe bound to both cellohexaose and xylohexaose, albeit with different affinities, but not to mannohexaose (Table 1). As expected, OC15 interacted 16 times more strongly with xylohexaose (34 × 10^3^ M^−1^) than with cellohexaose (2.1 × 10^3^ M^−1^). These affinity values are similar to those previously reported for CBM15 and confirm that the binding site of the recognition module of the OC15 probe is unaltered by the fusion with mOrange2 [45]. However, a small 1.7-fold increase is observed in the affinity constant of OC15 toward xylohexaose compared to CBM15 [45]. We attributed this increase to difference in experimental conditions in our study compared to those used previously. For instance, the sodium and calcium salt added to the binding buffer in our study may account for the observed difference. Such ions have also been observed in the crystallographic structure of CBM15 [45], although no biological relevance to their presence was given. We hypothesize that such a metallic ion may be important for the affinity and specificity of OC15 toward xylohexaose. We also found that OC15 bound weakly to cellohexaose but not to CMC (Fig. 2c). This result was unexpected, since the concentration of gel-embedded cellulose was 2.9 times higher than the *K*_d_ for cellohexaose (Table 1). This suggests that the bulkier carboxymethyl substitutions found in CMC may interfere with the affinity of the binding module of OC15 for cellulose. On the other hand, the presence of xylose moieties and/or xylan in a cellohexaose sample of high but imperfect purity (90 %) would also explain such an apparent contradiction.

**Table 1:** Affinity of the OC15 probe for various hexaoses as determined by ITC

Ligand	*K*_a_ × 10^3^ (M^−1^)	*K*_d_ (M)	n^a^
Xylohexaose	34 ± 0.2	2.938 × 10^−5^ ± 0.8	0.922 ± 0.1
Mannohexaose	NB^b^	–	–
Cellohexaose	2.1 ± 0.3	4.795 × 10^−4^ ± 0.1	1 ± 0.7

^a^ Number of ligand binding sites

^b^ No binding detected

### Comparing XPS, NREL/TP-510-42618, and OC15 methodologies for the detection of xylan

Pulps composed of a mixture of softwood and hardwood from an Eastern Canadian paper mill were used as lignocellulosic biomass for the formation of handsheets utilized in this study. Handsheets prepared from two grades of kraft pulp, UBKP and BKP, were investigated to determine differences in biopolymers content and their exposure at fiber surfaces. Kraft pulping and bleaching processes degrade and/or dissolve lignin. The removal of lignin through pulping increased access to xylan. In addition, removed xylan may redeposit onto the surface of cellulose fibers during pulping [26, 50–53]. The standard methods usually used for the detection of xylan are NREL/TP-510-42618 and XPS [35, 54–56]. These two approaches will be used and compared to our OC15 probe method.

The chemical composition of UBKP and BKP was determined by NREL/TP-510-42618 (Additional file 3) [35]. As expected, the pulp bleaching process decreased lignin by 2.3-fold without affecting the other biopolymers. Unfortunately, due to the nature of this technique, NREL/TP-510-42618 can only provide an overall bulk estimation of biopolymers content. It cannot detect small biopolymers changes nor measure variations of biopolymers exposition on the surface of fibers.

In contrast XPS has been extensively used for surface analysis of simple lignocellulosic biomasses to detect changes in surface coverage by cellulose, lignin, and extractives [54–56]. Elementary identification and bonding state discrimination are advantages associated to XPS analysis [37]. The C 1s band associated with lignocellulosic biomass which is monitored by XPS carries the most relevant information on surface polymers. C 1s spectrum has been suggested to result from the contribution of four different carbon functionalities: C1 (C–C, C–H, C=C), C2 (C–O or C–O–C), C3 (C=O or O–C–O), and C4 (O–C=O), which account for the chemical heterogeneity of paper fibers [54]. In cellulose, each glucose monomer harbors five C2 carbon atoms and one C3 carbon. Hemicelluloses are heterogeneous in their composition. Its monomers typically comprise fewer than five C2 carbon atoms, less than one C4 carbon atom and one C3 carbon atom. In contrast, lignin is more complex, having all four types of carbons with a greater contribution from C1 and C2 atoms [57–59]. In a typical fiber XPS analysis, C1 component mainly arises from lignin and extractives, while C2 signal is primarily associated to cellulose and hemicelluloses. C3 component is not easily assigned to a given polymer, as it is related to either carbonyl groups of lignin and extractives, or to carbon atoms bonded to two oxygen atoms in cellulose and hemicellulose [57–60]. C1 to C4 peaks were inferred from the deconvolution of the C 1s band for UBKP and BKP (Additional file 4). These deconvolutions were calculated using spectra as shown in Additional files 5 and 6. The bleaching process led to a 2.2-fold decrease in C1 functionality at the surface of the paper. This difference may be attributed to the removal of lignin from the surface as a normal consequence of bleaching. The decrease in lignin associated to C1 functionality is in line with the corresponding decrease in lignin measured by NREL/TP-510-42618 (Additional file 3). Interestingly, the bleaching process increased the C2 functionality by 1.1-fold, suggesting that cellulose and/or hemicellulose are slightly more exposed on the surface of BKP. The exposure of cellulose and hemicelluloses also increased C3 carbon detection by 1.2-fold. Due to the low concentration of carboxylic groups on the surface of kraft pulp, the C4 carbon functionality was minor and rather similar for either pulps. Like NREL/TP-510-42618, XPS analysis revealed the impact of the bleaching process on lignin. Moreover, XPS analyses suggested that lignin loss resulted in the increased exposure of cellulose and hemicellulose on the surface of BKP. Unfortunately, the C 1s spectra cannot distinguish cellulose from hemicellulose since both biopolymers possess similar carbon types. Moreover, XPS is not always reproducible due to the problems resulting from X-ray contamination and samples degradation [54, 61].

**Fig. 3:**
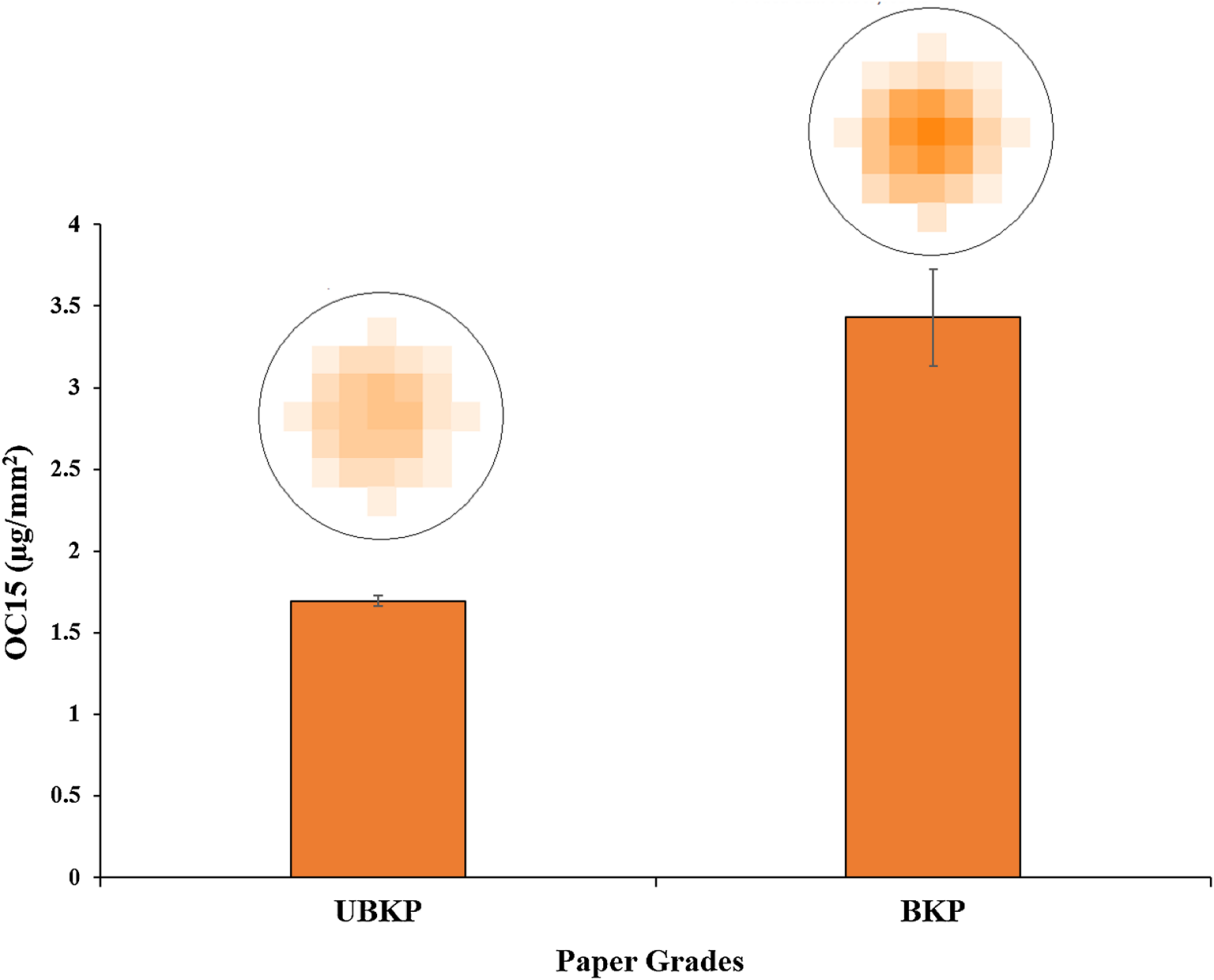
Quantification of OC15 binding to the surface of UBKP and BKP papers. UBKP and BKP paper punches were incubated with OC15 probe (0.5 µg/µL) for 1 h at room temperature under agitation. Three percent (w/v) milk (20 mM Tris–HCl, pH 7.5 with 20 mM NaCl and 5 mM CaCl_2_) was used to minimize paper auto-fluorescence and the non-specific binding of the OC15 probe. The *fluorescence* values were converted to OC15 (µg/mm^2^) using a standard curve (Additional file 9). The *inset* above each histogram columns represents the fluorescence intensity acquired by area scanning of the surface of each paper disc

Using OC15, we attempted to monitor the difference in xylan on the surface of UBKP and BKP papers resulting from the bleaching process. Complex lignocellulosic biomass fluoresces naturally when excited at the same wavelength as that for fluorescent protein mOrange2 *i.e.*, 549 nm (data not shown). This auto-fluorescence is mainly attributed to the lignin biopolymer found in kraft paper [62]. Thus, in order to minimize paper auto-fluorescence, we added a milk blocking step that also acted as a non-specific binding deterrent. Figure 3 describes the quantification of OC15 bound to the surface of UBKP and BKP papers. The bleached paper bound twice the amount of OC15 compared to the unbleached one, indicating that xylan exposure on the surface of kraft paper has increased after bleaching. This increase is fully compatible with the 2.3-fold decrease in lignin observed by chemical analysis, which was shielding xylan from surface detection before bleaching. This result confirms the loss of lignin that we measured using NREL/TP-510-42618 and XPS, demonstrating that our approach can efficiently detect the impact of the bleaching process on xylan. Therefore, introducing this xylan tracking approach as a quality control measurement would assuredly bolster the effectiveness of the lignocellulosic biomass process for selective as well as complete xylan removal.

### Monitoring xylan hydrolysis using NREL/TP-510-42618, XPS, and the OC15 probe

The effectiveness of xylan removal by xylanase hydrolysis of UBKP was investigated using NREL/TP-510-42618, XPS, and the OC15 probe. Chemical composition of untreated and xylanase-treated UBKP was analyzed (Additional file 7). As expected, xylanase treatment of pulp decreased xylose content by 1.7-fold without affecting lignin. The extractive content increased from 0.1 to 3.2 after xylanase treatment. This unexpected result is a consequence of the NREL lipids extraction methodology which consists into weighting the pulp before and after acetone solubilization of lipids [63]. Since the added xylanase accounts for 24.3 % of the pulp initial weight, its acetone removal from the pulp induces an apparent but false increase in lipids extractives.

We then studied the surface of untreated and xylanase-treated UBKP papers using XPS (deconvolution results described in Additional file 8). Overall, xylanase treatment of UBKP induced rather small variations in the carbon functionalities (C1 to C4). As such, the curve fitting component ascribed to C1 atoms slightly decreased (1.1-fold), indicating that the lignin biopolymer was marginally affected by xylose removal. Surprisingly, the C2 functionality associated to cellulose and hemicellulose was not altered by xylan hydrolysis. This result may be attributed to the exposure of cellulose on the fibers surface as a consequence of xylan removal by hydrolysis. The exposure of cellulose also increased C3 carbon functionality by 1.2-fold. The C4 carbon signal was minor and rather similar for either pulps. This study reveals that the impact of xylan digestion [which was clearly detected by NREL/TP-510-42618 (Additional file 7)] cannot be monitored unambiguously or directly by XPS.

**Fig. 4:**
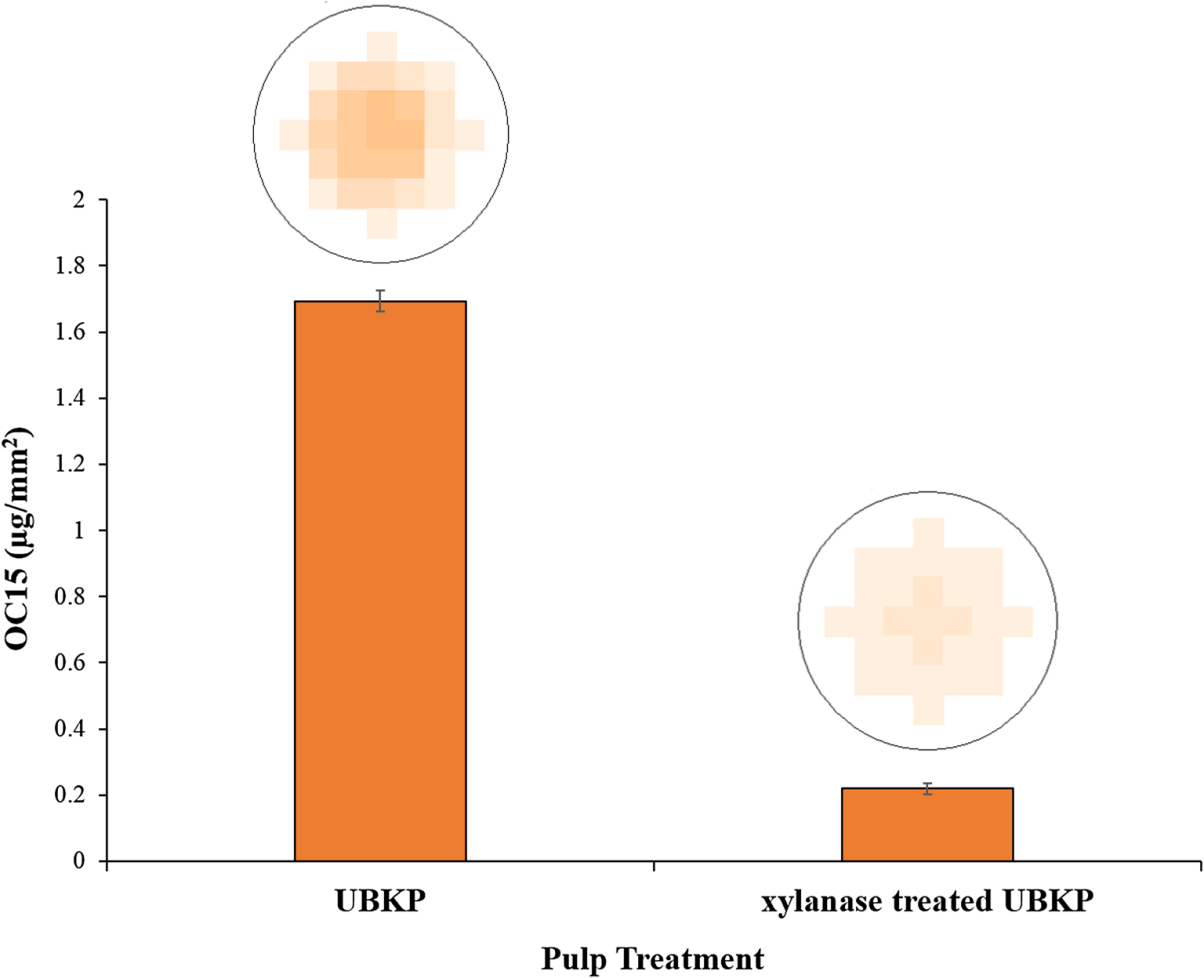
Quantification of OC15 binding to the surface of untreated and xylanase-treated UBKP papers. Pulps were incubated with or without the xylanase (500 U/g of pulp) for 1 h (pH 6) at room temperature under continuous agitation (150 rpm). Untreated UBKP and xylanase-treated UBKP paper discs were incubated with OC15 probe (0.5 µg/µL) for 1 h at room temperature under agitation. Three percent (w/v) milk (20 mM Tris–HCl, pH 7.5 with 20 mM NaCl and 5 mM CaCl_2_) was used to minimize paper auto-fluorescence and the non-specific binding of the OC15 probe. The *fluorescence* values were converted to OC15 (µg/mm^2^) using a standard curve (Additional file 9). The *inset* above each histogram columns represents the fluorescence intensity acquired by area scanning of the surface of each paper disc

**Fig. 5:**
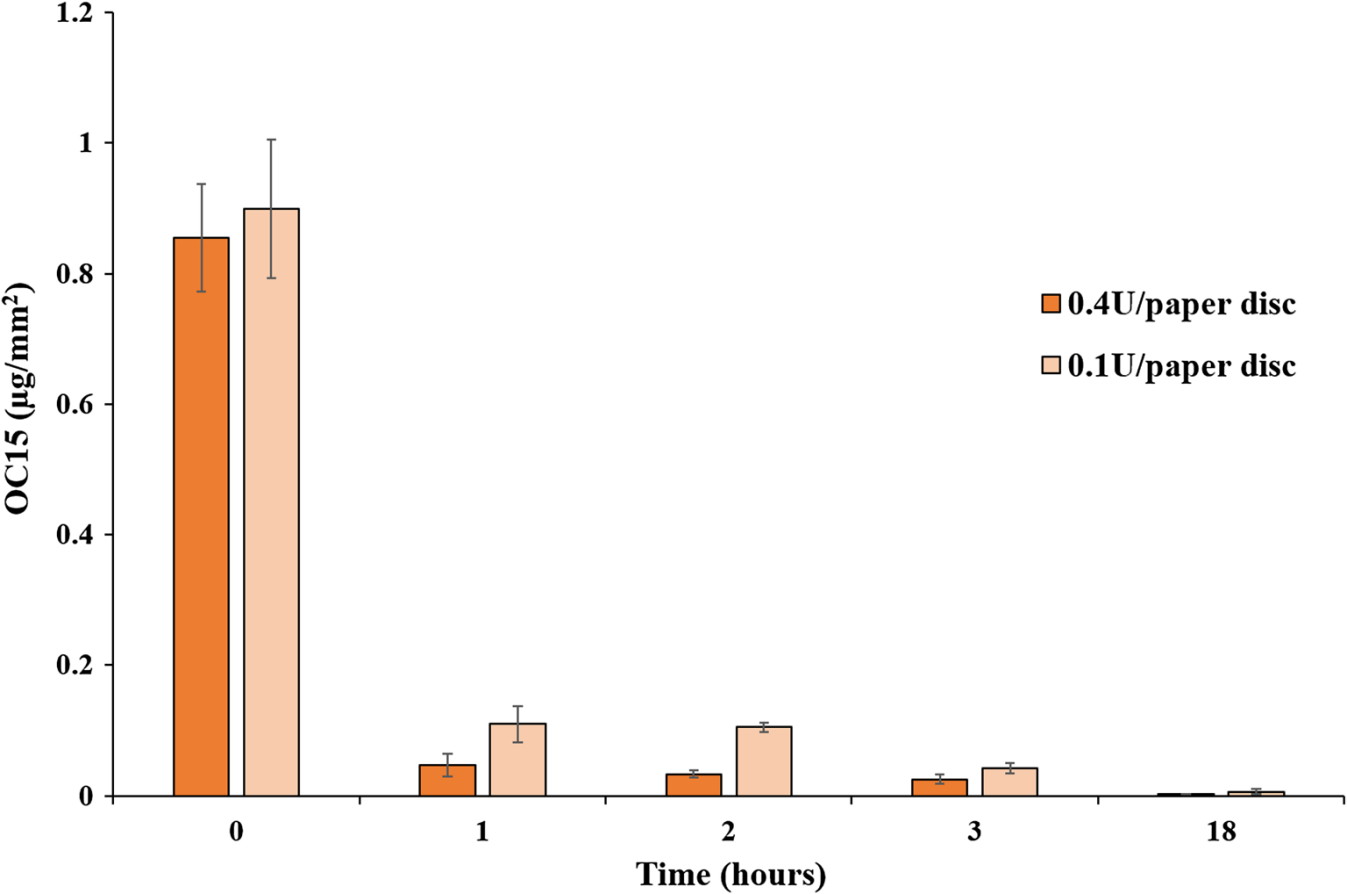
Tracking xylanase hydrolysis of UBKP using OC15 probe. Paper discs were incubated with xylanase (0.4 U/paper disc and 0.1 U/paper disc) for up to 18 h (pH 6) at room temperature under continuous agitation (150 rpm). Untreated UBKP and xylanase-treated UBKP paper discs were incubated with OC15 probe (0.06 µg/µL) for 1 h at room temperature under agitation. Three percent (w/v) milk (20 mM Tris–HCl, pH 7.5 with 20 mM NaCl and 5 mM CaCl_2_) was used to minimize paper auto-fluorescence and non-specific binding of the OC15 probe. The *fluorescence* values were converted to OC15 (µg/mm^2^) using a standard curve (Additional file 9)

The impact of xylanase on xylan at the surface of UBKP paper discs was investigated using OC15 probe. A decrease in xylan was clearly indicated by the 7.7-fold decrease in OC15 binding after xylanase treatment (Fig. 4). The use of OC15 probe confirmed the loss of xylan suggested by chemical analysis (NREL/TP-510-42618) with the distinction that OC15 specifically probes fiber surface. We also studied the binding of OC15 to xylanase-treated UBKP paper discs as a function of time and enzyme dosages (0.4 vs 0.1 U). The xylanase digestions were performed on paper discs glued in 96-wells microtiter plates over an 18 h incubation period at room temperature. Figure 5 reveals that after 1 h a significant removal of surface xylan was detected. Xylan was reduced 8.2-fold by 0.1 xylanase units and 17-fold when 0.4 units were used. The complete removal of xylan was detected after 18 h of incubation (0.4 unit dosage). OC15 binding responded proportionally to enzyme load and allowed monitoring xylanase treatment kinetics. This high throughput method enables the screening for optimal xylanase hydrolysis conditions, necessary for removal of xylan from kraft paper. We predict that OC15 usefulness is not limited to kraft paper analysis, but should include optimization of any biomass process for which surface xylan is determinant.

## Conclusion

Monitoring the impact of mechanical, chemical, and enzymatic modifications of biopolymers found in lignocellulosic biomasses is a complex endeavor. The currently available methods for chemical composition analysis of biopolymers in pulp, such as NREL/TP-510-42618, are able to only quantify bulk xylan but give no information on biopolymers surface exposition. On the other hand, XPS, while being highly sensitive, cannot unambiguously monitor changes in surface xylan since cellulose and hemicellulose share similar C 1s carbon functionalities. These standard methods are poorly adapted to important problematics associated with biofuels and pulp and paper industries. To address those issues, we developed a novel xylan detection approach that is sensitive, specific, reproducible, rapid (hundreds of samples analyzed in less than 4 h), high throughput, cost-effective, and that requires minimal specialized equipment. This approach involves solely the utilization of a two-domain probe, OC15, which harnesses the specific xylan recognition power of CBM15 and the high sensitivity of mOrange2 fluorescence emission.

Our results demonstrate that OC15 enables the specific tracking of chemical and enzymatic-induced variations of xylan on the surface of kraft pulps. In addition, we demonstrated here that our approach can be readily adapted to a high throughput format (tests were performed in multiwell plates and analyzed with a plate reader). We believe that this tracking approach could perform various functions, such as (1) fine-tuning the conditions surrounding the mechanical and enzymatic removal of xylan; (2) decreasing costs associated with lignocellulosic biomass processes; (3) expanding our understanding of biofuels and papermaking productions; (4) correlating surface xylan with performances of the relevant lignocellulosic products; and (5) improving the productivity of large-scale operations. This study testifies to the incredible versatility of CBMs as spearheads of innovations which can successfully tackle biotechnological challenges.

## Methods

### Chemicals and strains

Unless otherwise noted, all chemicals were reagent grade and purchased from Sigma-Aldrich or Fisher Scientific. *Escherichia coli* XL10 cells (Agilent Technologies) were used for all DNA manipulations while *E. coli* BL21-Gold(DE3)pLysS competent cells (Agilent Technologies) were used for recombinant protein expression. *Trichoderma viride* xylanase from glycoside hydrolase (GH) family 11 (cat no. 95595; Sigma-Aldrich) was used for the digestion of lignocellulosic biomasses. Xylanase activity was 16.57 U/g.

### Construction of pET11a-mOrange2-CBM15 expression vector

The CBM15 gene (xylan binding domain) was cloned into the C-terminal end of the mOrange2 gene (detection domain) in a pET11a vector. Briefly, *Cellvibrio japonicus* CBM15 (GenBank Accession Z48928) was synthetized by GenScript and provided as part of the pUC57-CBM15 vector. In order to insert the *Bsr*GI and *Bam*HI restriction sites (underlined) at each end of CBM15, we amplified the gene using forward (5′-*TGTACA*AGGGTGTCGCTGCCAGC-3′) and reverse primers (5′-*GGATCC*TTAATTGGCTGAATAGGCTTCC-3′). The resulting PCR product was then purified using Qiagen MinElute PCR purification kit. In addition, the mOrange2 gene was excised from the pmOrange2 vector (Clontech) using a *Dra*III and *Bam*HI double digestion and inserted into the corresponding sites of pET11a vector. Finally, the double *Bsr*GI and *Bam*HI digestion of CBM15 was purified and inserted into the corresponding site of the pET11a-mOrange2 vector, resulting into the pET11a-mOrange2-CBM15 expression vector. At each step, the constructs were sequenced to ascertain the integrity and fidelity of the products DNA sequence.

### Expression and purification of OC15 probe

*Escherichia coli* BL21-Gold(DE3)pLysS cells (Agilent Technologies) bearing the OC15 expression plasmid were grown at 37 °C and 200 rpm in Luria-Bertani broth containing 100 μg/mL of ampicillin. Induction of recombinant protein expression was performed by the addition of 500 μM IPTG (Thermo Fisher Scientific) to mid-log-phase cells (O.D._600nm_ of 0.6–0.8) and subsequent incubation for 18 h at 25 °C. Cells were then harvested and kept at −80 °C. Thawed cell pellets were resuspended in 50 mM sodium phosphate pH 8 containing 300 mM NaCl, 2 mM imidazole, 1 mM PMSF, and then lysed by sonication using six cycles of 60 s (Branson Ultrasonics Corporation) at 200 W. Clarification of lysate was achieved by centrifuging at 10,000*g* for 30 min at 4 °C. The protein of interest was purified by affinity chromatography over a HisPrep FF 16/10 column (GE Healthcare Life Sciences) equilibrated in 50 mM sodium phosphate buffer pH 8.0 containing 300 mM NaCl and 10 mM imidazole. After washing with ten column volumes of buffer, the desired protein was eluted using a gradient of imidazole (10–250 mM) in 50 mM sodium phosphate pH 8.0 buffer containing 300 mM NaCl. A final purification step was performed using a Superdex 200 HR 16/50 column (GE Healthcare Life Sciences) in 50 mM Tris–HCl pH 7.5 buffer containing 300 mM NaCl to insure its homogenous purity. The purified probe was then dialyzed in a 20 Tris–HCl pH 7.5 buffer containing 20 mM NaCl and 5 mM CaCl_2_ at 4 °C and concentrated using a 10K Macrosep Advance centrifugal device (Pall Corporation). Concentrated protein solutions were stored at −80 °C using flash freezing. Protein purity (expected mass 44.68 kDa) was verified by SDS-PAGE. The amount of protein was quantified by the Bradford method [64].

### Affinity gel electrophoresis (AGE)

AGE was used for qualitative assessment of OC15 (10 µg) specificity toward selected ligands. The experiment was performed as described elsewhere [49, 65], by adding 0.5 % (w/v) of beechwood xylan (Sigma-Aldrich), carboxymethyl cellulose (CMC) (Sigma-Aldrich), and galactomannan (Megazyme) to a native, 12 % polyacrylamide gel. Bovine serum albumin (BSA) (10 µg/well) was used as negative control since it has no affinity toward carbohydrates [49].

### Isothermal titration calorimetry (ITC)

ITC was employed to measure the affinity of the OC15 probe toward selected hexaoses (Megazyme). Cellohexaose, xylohexaose, and mannohexaose were reconstituted in a 20-mM Tris–HCl pH 7.5 buffer which contained 20 mM NaCl and 5 mM CaCl_2_. The purified OC15 probe was also dialyzed into that same buffer. All experiments were performed with a Nano ITC microcalorimeter (TA Instruments) operated at 25 °C with a stirring rate set of 250 rpm. Pre-equilibrated solutions of probe (200 μM) and hexaoses (5 mM) were used for each assay. The control experiments were based on titrations of hexaoses into the buffer and buffer into the OC15 probe. Each experiment consisted of 25 injections of 2 μL hexaose into the probe solution, with an interval of 130 s between injections. All experiments were performed in triplicates. Data were analyzed and fitted using the NanoAnalyze software v2.3.6 (TA Instruments).

### Pulp characterization

The kraft pulps used for this study were provided by an Eastern Canadian pulp and paper company. The kraft pulping was performed using a mixture of softwood and hardwood. Two different grades of pulps, unbleached kraft pulp (UBKP) and bleached kraft pulp (BKP), were used. The cellulose, hemicellulose, and lignin contents of these pulps were analyzed according to NREL/TP-510-42618 protocol [35]. The hydrolyzed monosaccharides contents of the pulps (10 µL injection) were determined by ion chromatography (ICS-5000, Dionex) and detection was performed using an electrochemical detection cell (combined pH-Ag/AgCl reference electrode). Each experiment was conducted at 40 °C with 1 mL/min isocratic elution of NaOH (1 mM) on a Dionex CarboPac SA10 (250 × 4 mm) column coupled with a Dionex CarboPac PA100 (50 × 4 mm) guard column. Data analysis was performed using Dionex Chromeleon 7 software.

### Handsheets preparation

UBKP and BKP were used as lignocellulosic substrates for the preparation of handsheets and paper discs. Handsheets (basis weight of 60 ± 2 g/m^2^) were prepared from pulp according to TAPPI standard method T 205 sp-02. Prior to testing, the handsheets were conditioned for 24 h at room temperature and 50 % of relative humidity according to TAPPI method T 402 sp-03 [66]. These handsheets were then used for the preparation of the paper punches. The paper punches are defined as paper discs having diameter of 3 mm.

### Xylanase digestion of unbleached kraft pulp

Xylanase digestion of UBKP was done according to Li et al. [66]. Briefly, the presoaked, disintegrated pulp at 2 % consistency was incubated 1 h at pH 6 and room temperature under continuous agitation (150 rpm), with or without xylanase (500 U/g of pulp). The reactions were stopped by a 15-min incubation on ice. The pulp was then used for chemical composition analysis (NREL/TP-510-42618) and handsheets formation.

### X-ray photoelectron spectroscopy (XPS)

The 300 Watts monochromatic Al K-α radiation source originating from an AXIS-ULTRA apparatus (KRATOS ANALYTICAL) was used to study xylan. The analyser was set in the constant pass energy mode, the lens set to the hybrid configuration (both magnetic and electrostatic lenses), and the electrostatic lens aperture in the slot position. This configuration provided the highest sensitivity for scanning 700 × 300 µm area. Three different spots were analyzed to obtain an average. The pressure of the system was set at 10^−8^ Torr. Elemental analysis of the surface area was performed by recording survey spectra at 160 eV with energy increment of 1 eV per channel. High resolution spectra were recorded at 20 eV with energy increment of 0.05 eV. This setup gave an overall instrumental resolution of 0.6 eV as measured on Ag3d_5/2_. Analyses of the peak decompositions were performed using the CasaXPS software.

### Xylan tracking on the surface of papers using the OC15 probe

All fluorescence readings were acquired at room temperature with a Synergy Mx microplate reader (BioTek) using the area scanning feature (3 × 3) with the top detection height set at 4.5 mm and the filters bandwidth at 9 mm. The excitation and emission wavelengths were set at 549 and 568 nm for the OC15 probe. Each experiment was done in triplicates. Two different grades of kraft pulps, unbleached (UBKP) and bleached (BKP), were investigated regarding their xylan content. The following method is a modified high throughput version of the methodology described by Knox [38]. Hence, it was performed into 96-well black microtiter plates (Corning), where each well contained a 3 mm diameter paper disc obtained from 60 g/m^2^ handsheet. The discs were glued to the bottom of each well and first incubated for 1 h at room temperature with agitation in 3 % (w/v) milk (20 mM Tris–HCl, pH 7.5 with 20 mM NaCl, and 5 mM CaCl_2_) to minimize paper auto-fluorescence and the non-specific binding of the OC15 probe. Milk excess was then removed with 3 × 5 washing steps using the assay buffer. At this stage, the fluorescence intensity of the paper discs was measured and referred as to blank fluorescence. The specific binding of the OC15 probe to the surface of the paper discs was initiated by adding 0.5 µg/µL of the OC15 probe in assay buffer to each well. After a 1 h incubation at room temperature under agitation, the excess and/or non-specifically bound probe were removed by 3 × 5 min washes with buffer that also contained 0.05 % (v/v) of Tween 20. The residual fluorescence intensity associated with the specific detection of xylan was then recorded. Quantification of the bound OC15 probe was achieved by subtracting the value of the mean blank fluorescence from the mean residual fluorescence obtained for each well. These fluorescence values were then converted into µg/mm^2^ using the appropriate standard curves (Additional file 9) and the surface area of paper discs.

## Additional files

10.1186/s13068-016-0486-1 SDS-PAGE analysis of the OC15 probe purified by affinity chromatography. The expected molecular weight of the OC15 fusion protein is 44.68 kDa. A 12 % polyacrylamide gel was used for SDS-PAGE analysis. Well M: Precision plus protein standards (5 µg). Well OC15: Purified OC15 probe (10 µg).
10.1186/s13068-016-0486-1 Isothermal calorimetric titration of the OC15 probe with xylohexaose. Top panel: Typical ITC experiment carried out by adding 25 injections of 2 μL xylohexaose (5 µM) into the OC15 probe (200 mM) solution, with an interval of 130 s between each injection. Bottom panel: Heat release per mole of xylohexaose as a function of xylohexaose/OC15 molar ratio. The titration was performed at 25 °C in a 20 mM Tris-HCl pH 7.5 buffer which contained 20 mM NaCl and 5 mM CaCl_2_. Injectant: xylohexaose.
10.1186/s13068-016-0486-1 Chemical composition of UBKP and BKP determined by NREL/TP-510-42618. UBKP: Unbleached kraft pulp. BKP: Bleached kraft pulp.
10.1186/s13068-016-0486-1 XPS analysis of UBKP and BKP. Results include O/C ratios and contributions (%) from each carbon type (C1-C4) to curve fitting of the C 1s peak measured by low- and high-resolution XPS. UBKP: Unbleached kraft pulp. BKP: Bleached kraft pulp.
10.1186/s13068-016-0486-1 Low-resolution XPS spectrum of UBKP surface. UBKP: unbleached kraft pulp. Unextracted pulp samples were analysed.
10.1186/s13068-016-0486-1 Deconvolution of high-resolution XPS spectrum of UBKP. UBKP: unbleached kraft pulp. Unextracted pulp samples were analysed.
10.1186/s13068-016-0486-1 Chemical composition of untreated and xylanase-treated UBKP determined by NREL/TP-510-42618. UBKP: Unbleached kraft pulp.
10.1186/s13068-016-0486-1 XPS analysis of UBKP and xylanase-treated UBKP. Results include O/C ratios and contributions (%) from each carbon type (C1-C4) to curve fitting of the C 1s peak measured by low- and high-resolution XPS. UBKP: Unbleached kraft pulp.
10.1186/s13068-016-0486-1 Standard curve for the conversion of fluorescence intensity into µg of OC15 probes. The excitation and emission wavelengths were set at 549 and 568 nm respectively.

## Abbreviations

AGE: affinity gel electrophoresis
BSA: bovine serum albumin
BKP: bleached kraft pulp
CAZy: carbohydrate active enzymes
CBMs: carbohydrate-binding modules
CBM15: family 15 carbohydrate-binding module
CMC: carboxymethyl cellulose
GH: glycoside hydrolase
IPTG: isopropyl-β-D-thiogalactopyranoside
ITC: isothermal titration calorimetry
LB: Luria–Bertani
mAbs: monoclonal antibodies
mOrange2: mono-orange2
NREL: national renewable energy laboratory
OC15: mono-orange2 fluorescent protein linked to a family 15 carbohydrate-binding module
PCR: polymerase chain reaction
SDS-PAGE: sodium dodecyl sulfate-polyacrylamide gel electrophoresis
UBKP: unbleached kraft pulp
XPS: X-ray photoelectron spectroscopy
