# Supplementary material for: Specific tracking of xylan using fluorescent-tagged carbohydrate-binding module 15 as molecular probe
Source: Biotechnol Biofuels. 2016 Mar 25;9:74. doi: 10.1186/s13068-016-0486-1 (PMC4807533; doi:10.1186/s13068-016-0486-1)
Supplement: Supplementary file 1 — 10.1186/s13068-016-0486-1 SDS-PAGE analysis of the OC15 probe purified by affinity chromatography. The expected molecular weight of the OC15 fusion protein is 44.68 kDa. A 12 % polyacrylamide gel was used for SDS-PAGE analysis. Well M: Precision plus protein standards (5 µg). Well OC15: Purified OC15 probe (10 µg). [file 13068_2016_486_MOESM1_ESM.docx]

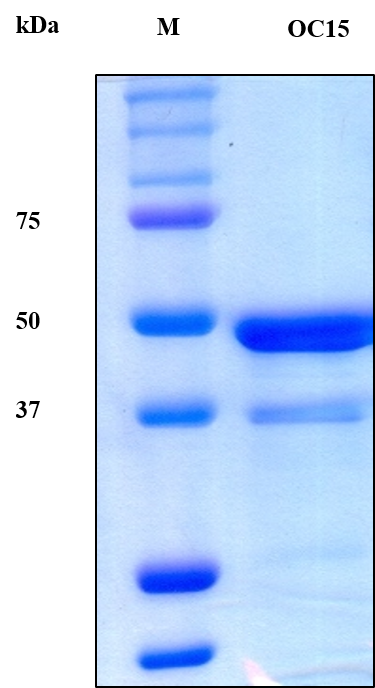


**Additional file 1: Figure S1. SDS-PAGE analysis of the OC15 probe purified by affinity chromatography.** The expected molecular weight of the OC15 fusion protein is 44.68 kDa. A 12% polyacrylamide gel was used for SDS-PAGE analysis. Well M: Precision plus protein standards (5 µg). Well OC15: Purified OC15 probe (10 µg).
