# Supplementary material for: Specific tracking of xylan using fluorescent-tagged carbohydrate-binding module 15 as molecular probe
Source: Biotechnol Biofuels. 2016 Mar 25;9:74. doi: 10.1186/s13068-016-0486-1 (PMC4807533; doi:10.1186/s13068-016-0486-1)
Supplement: Supplementary file 2 — 10.1186/s13068-016-0486-1 Isothermal calorimetric titration of the OC15 probe with xylohexaose. Top panel: Typical ITC experiment carried out by adding 25 injections of 2 μL xylohexaose (5 µM) into the OC15 probe (200 mM) solution, with an interval of 130 s between each injection. Bottom panel: Heat release per mole of xylohexaose as a function of xylohexaose/OC15 molar ratio. The titration was performed at 25 °C in a 20 mM Tris-HCl pH 7.5 buffer which contained 20 mM NaCl and 5 mM CaCl2. Injectant: xylohexaose. [file 13068_2016_486_MOESM2_ESM.docx]

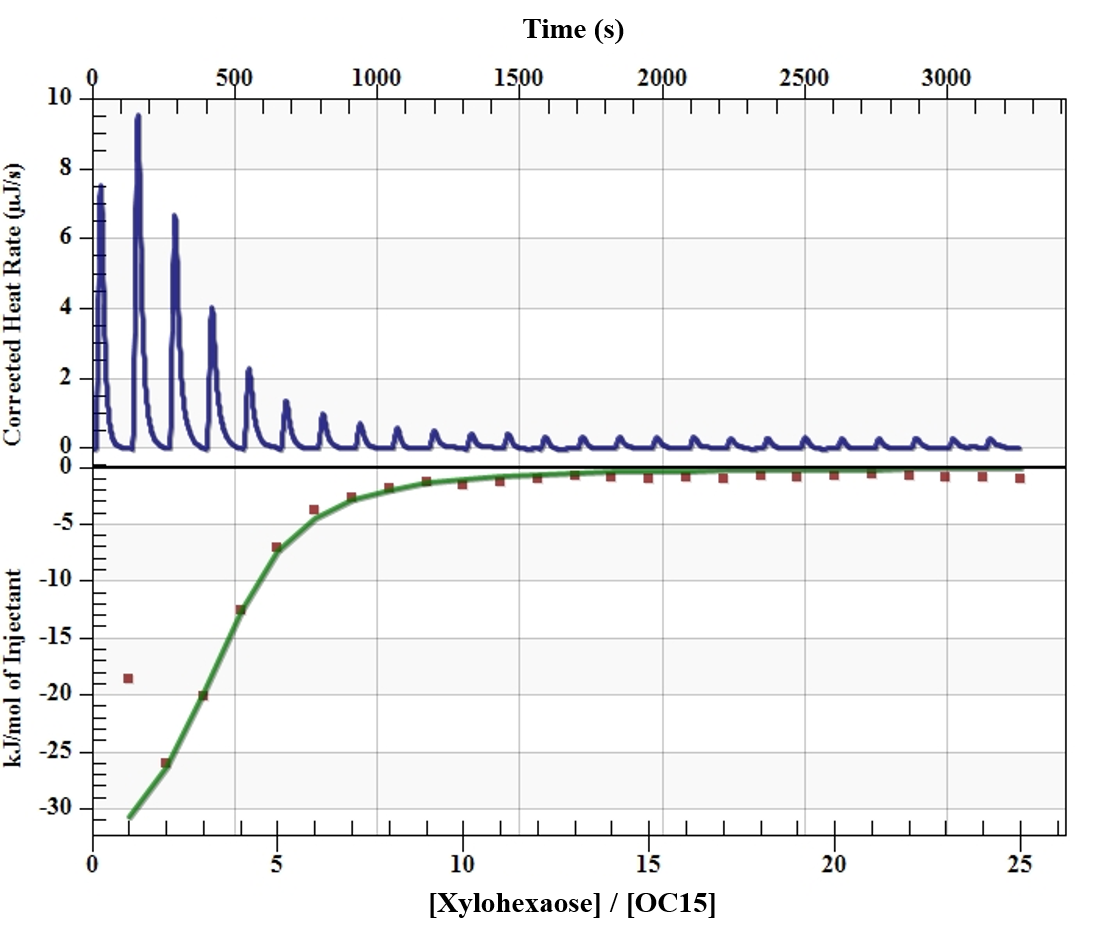
**Additional file 2: Figure S2. Isothermal calorimetric titration of the OC15 probe with xylohexaose.** Top panel: Typical ITC experiment carried out by adding 25 injections of 2 μL xylohexaose (5 µM) into the OC15 probe (200 mM) solution, with an interval of 130 seconds between each injection. Bottom panel: Heat release per mole of xylohexaose as a function of xylohexaose/OC15 molar ratio. The titration was performed at 25°C in a 20 mM Tris-HCl pH 7.5 buffer which contained 20 mM NaCl and 5 mM CaCl_2_. Injectant: xylohexaose.
