# Supplementary material for: Specific tracking of xylan using fluorescent-tagged carbohydrate-binding module 15 as molecular probe
Source: Biotechnol Biofuels. 2016 Mar 25;9:74. doi: 10.1186/s13068-016-0486-1 (PMC4807533; doi:10.1186/s13068-016-0486-1)
Supplement: Supplementary file 3 — 10.1186/s13068-016-0486-1 Chemical composition of UBKP and BKP determined by NREL/TP-510-42618. UBKP: Unbleached kraft pulp. BKP: Bleached kraft pulp. [file 13068_2016_486_MOESM3_ESM.docx]

**Additional file 3: Table S1. Chemical composition of UBKP and BKP determined by NREL/TP-510-42618.** UBKP: Unbleached kraft pulp. BKP: Bleached kraft pulp.

| **Compound** | **UBKP**  **(%)** | **BKP**  **(%)** |
| --- | --- | --- |
| Extractives | 0.3 ± 0.01 | 0.3 ± 0.01 |
| Lignin | 4.4 ± 0.10 | 1.9 ± 0.05 |
| Glucose | 80.8 ± 0.92 | 83.6 ± 1.97 |
| Xylose | 7.9 ± 0.63 | 8.5 ± 0.76 |
| Mannose | 6.7 ± 0.67 | 7.0 ± 0.65 |
| Galactose | 0.0 ± 0.00 | 0.0 ± 0.00 |
| Arabinose | 0.0 ± 0.00 | 0.0 ± 0.00 |
