# Supplementary material for: Specific tracking of xylan using fluorescent-tagged carbohydrate-binding module 15 as molecular probe
Source: Biotechnol Biofuels. 2016 Mar 25;9:74. doi: 10.1186/s13068-016-0486-1 (PMC4807533; doi:10.1186/s13068-016-0486-1)
Supplement: Supplementary file 4 — 10.1186/s13068-016-0486-1 XPS analysis of UBKP and BKP. Results include O/C ratios and contributions (%) from each carbon type (C1-C4) to curve fitting of the C 1s peak measured by low- and high-resolution XPS. UBKP: Unbleached kraft pulp. BKP: Bleached kraft pulp. [file 13068_2016_486_MOESM4_ESM.docx]

**Additional file 4: Table S2. XPS analysis of UBKP and BKP.** Results include O/C ratios and contributions (%) from each carbon type (C1-C4) to curve fitting of the C 1s peak measured by low- and high-resolution XPS. UBKP: Unbleached kraft pulp. BKP: Bleached kraft pulp.

| **Functionality** | **UBKP**  **(%)** | **BKP**  **(%)** |
| --- | --- | --- |
| **O/C*** | 0.61 ±0.04 | 0.64 ±0.01 |
| **C1** | 20.1 ±0.1 | 9.1 ±0.5 |
| **C2** | 63.0 ±1.6 | 71.6 ±1.9 |
| **C3** | 15.8 ±0.1 | 18.3 ±0.4 |
| **C4** | 1.1 ±0.3 | 1.0 ± 0.7 |

Spectra were taken from unextracted pulp samples.

*****Low-resolution XPS spectra was used to obtain the oxygen and carbon percentage in order to ascertain that the O/C ratio does not vary as a function of chemical treatment. The bleaching process did not change the overall percentage of oxygen and carbon.
