# Supplementary material for: Specific tracking of xylan using fluorescent-tagged carbohydrate-binding module 15 as molecular probe
Source: Biotechnol Biofuels. 2016 Mar 25;9:74. doi: 10.1186/s13068-016-0486-1 (PMC4807533; doi:10.1186/s13068-016-0486-1)
Supplement: Supplementary file 6 — 10.1186/s13068-016-0486-1 Deconvolution of high-resolution XPS spectrum of UBKP. UBKP: unbleached kraft pulp. Unextracted pulp samples were analysed. [file 13068_2016_486_MOESM6_ESM.docx]

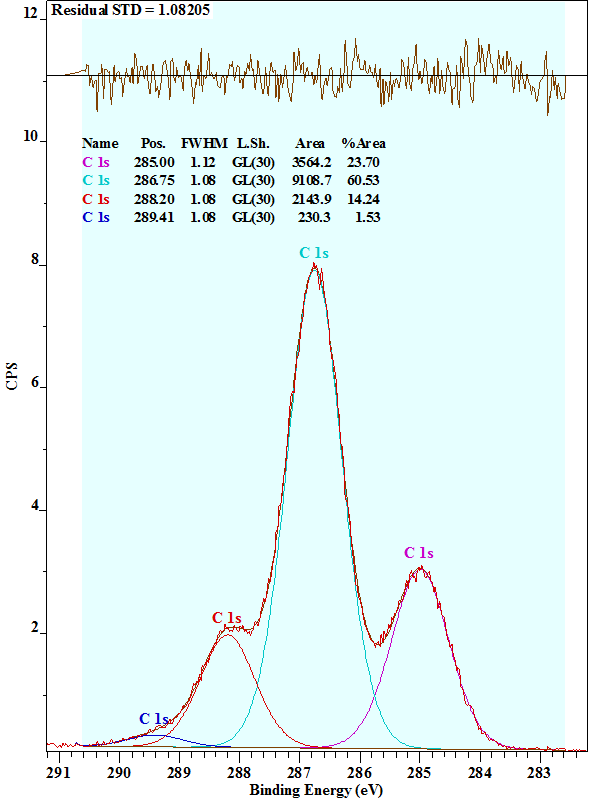


**Additional file 6: Figure S4. Deconvolution of high-resolution XPS spectrum of UBKP.** UBKP: unbleached kraft pulp**.** Unextracted pulp samples were analysed.
