# Supplementary material for: Specific tracking of xylan using fluorescent-tagged carbohydrate-binding module 15 as molecular probe
Source: Biotechnol Biofuels. 2016 Mar 25;9:74. doi: 10.1186/s13068-016-0486-1 (PMC4807533; doi:10.1186/s13068-016-0486-1)
Supplement: Supplementary file 7 — 10.1186/s13068-016-0486-1 Chemical composition of untreated and xylanase-treated UBKP determined by NREL/TP-510-42618. UBKP: Unbleached kraft pulp. [file 13068_2016_486_MOESM7_ESM.docx]

**Additional file 7: Table S3. Chemical composition of untreated and xylanase-treated UBKP determined by NREL/TP-510-42618.** UBKP: Unbleached kraft pulp.

| **Compound** | **UBKP**  **(%)** | **Xylanase-treated UBKP (%)** |
| --- | --- | --- |
| Extractives | 0.1 ± 0.04 | 3.2 ± 0.02 |
| Lignin | 4.6 ± 0.07 | 4.6 ± 0.10 |
| Glucose | 81.0 ± 0.54 | 81.8 ± 0.73 |
| Xylose | 8.1 ± 0.28 | 4.8 ± 0.25 |
| Mannose | 2.9 ± 0.37 | 4.0 ± 0.19 |
| Galactose | 0.4 ± 0.04 | 0.4 ± 0.04 |
| Arabinose | 0.7 ± 0.01 | 0.5 ± 0.08 |
