# Supplementary material for: Specific tracking of xylan using fluorescent-tagged carbohydrate-binding module 15 as molecular probe
Source: Biotechnol Biofuels. 2016 Mar 25;9:74. doi: 10.1186/s13068-016-0486-1 (PMC4807533; doi:10.1186/s13068-016-0486-1)
Supplement: Supplementary file 8 — 10.1186/s13068-016-0486-1 XPS analysis of UBKP and xylanase-treated UBKP. Results include O/C ratios and contributions (%) from each carbon type (C1-C4) to curve fitting of the C 1s peak measured by low- and high-resolution XPS. UBKP: Unbleached kraft pulp. [file 13068_2016_486_MOESM8_ESM.docx]

**Additional file 8: Table S4. XPS analysis of UBKP and xylanase-treated UBKP.** Results include O/C ratios and contributions (%) from each carbon type (C1-C4) to curve fitting of the C 1s peak measured by low- and high-resolution XPS. UBKP: Unbleached kraft pulp**.**

| **Functionality** | **UBKP**  **(%)** | **Xylanase-treated UBKP**  **(%)** |
| --- | --- | --- |
| **O/C*** | 0.52 ± 0.01 | 0.55 ± 0.04 |
| **C1** | 24.1 ± 0.5 | 21.6 ± 0.4 |
| **C2** | 61 ± 2.3 | 61 ± 1.4 |
| **C3** | 13.8 ± 0.1 | 16.0 ± 0.9 |
| **C4** | 1.5 ± 0.4 | 1.7 ± 0.2 |

Spectra were taken from unextracted pulp samples.

*Low-resolution XPS spectra was used to obtain the oxygen and carbon percentage in order to ascertain that the O/C ratio does not vary as a function of chemical treatment. The xylanase treatment did not change the overall percentage of oxygen and carbon.
