# Supplementary material for: Specific tracking of xylan using fluorescent-tagged carbohydrate-binding module 15 as molecular probe
Source: Biotechnol Biofuels. 2016 Mar 25;9:74. doi: 10.1186/s13068-016-0486-1 (PMC4807533; doi:10.1186/s13068-016-0486-1)
Supplement: Supplementary file 9 — 10.1186/s13068-016-0486-1 Standard curve for the conversion of fluorescence intensity into µg of OC15 probes. The excitation and emission wavelengths were set at 549 and 568 nm respectively. [file 13068_2016_486_MOESM9_ESM.docx]

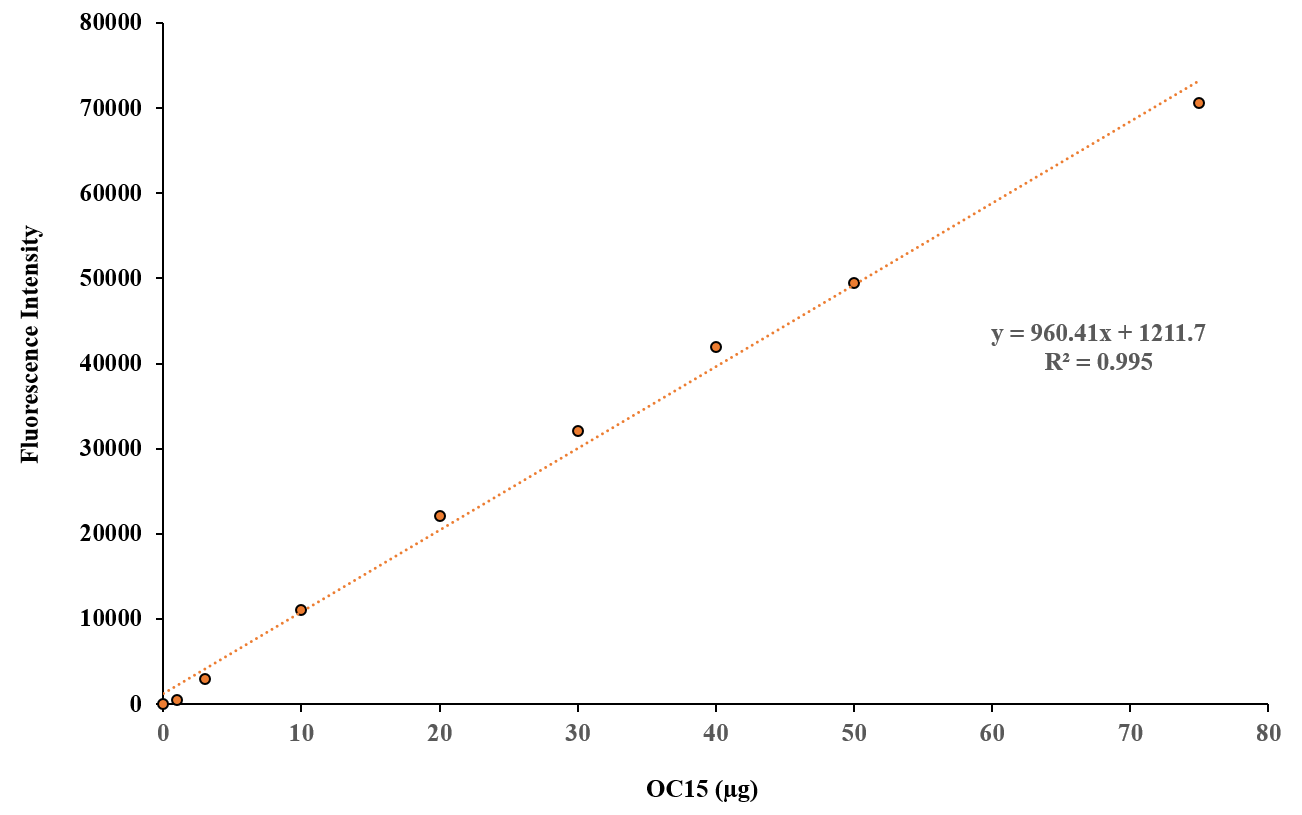


**Additional file 9: Figure S5. Standard curve for the conversion of fluorescence intensity into µg of OC15 probes.** The excitation and emission wavelengths were set at 549 and 568 nm respectively.
